# Trauma team conformation in a war-influenced middle-income country in South America: is it possible?

**DOI:** 10.1186/s12245-020-00297-7

**Published:** 2020-07-14

**Authors:** Sandra Carvajal, Francisco L Uribe-Buritica, Ana Maria Ángel-Isaza, María Camila López-Girón, Andres González, Julian Chica, Manuel Benitez, Alberto F García

**Affiliations:** 1 Fundación Valle del Lili, Emergency Department, Fundación Valle del Lili, Cra 98 No 18-49, Cali, 760032 Colombia; 2 Clinical Research Center, Centro de Investigaciones Clínicas, Fundación Valle del Lili, Cra 98 No 18-49, Cali, 760032 Colombia; 3 Universidad Icesi, Undergraduate Medicine Department, Universidad Icesi, Cl. 18 #122-135, Cali, Valle del Cauca Colombia; 4 Fundación Valle del Lili, Surgery Department, Fundación Valle del Lili, Cra 98 No 18-49, Cali, 760032 Colombia; 5 Fundación Valle del Lili, Intensive Care Unit, Fundación Valle del Lili, Cra 98 No 18-49, Cali, 760032 Colombia

**Keywords:** Trauma severity indices, Trauma centers, Multiple trauma, Injuries, Advanced Trauma Life Support Care

## Abstract

**Introduction:**

Trauma teams (TTs) improve outcomes in trauma patients. A multidisciplinary TT was conformed in September 2015 in a tertiary level I trauma university hospital in southwestern Colombia, a middle-income war-influenced country.

**Objective:**

To evaluate the impact of a TT in admission-tomography and admission-surgery times as well as mortality in a tertiary center university hospital in a middle-income country war-influenced country.

**Material and methods:**

Retrospective analytical study. Patients older than 17 years admitted to the emergency room 15 months prior and 15 months after the TT implementation were included. Patients prior to the TT implementation were taken as controls. No exclusion criteria. Four hundred sixty-four patients were included, 220 before the TT implementation (BTT) and 244 after (ATT). Demographic data, trauma characteristics, admission-tomography, and admission-surgery time interval as well as mortality were recorded. Requirement of CT scan or surgery was based on physician decision. The analysis was made on Stata 15.1®. Categorical variables were described as quantities and proportions, and continuous variables as mean and standard deviation or median and interquartile range (IQR). Categorical variables were compared using *χ*^2^ or Fisher’s test and continuous variables using Student’s *T* test or Wilcoxon-Mann-Whitney. A multiple logistic regression model was created to evaluate the impact of being treated in the ATT group on mortality, adjusted by age, trauma severity, and physiological response upon admission.

**Results:**

The admission-tomography time interval was 56 min (IQR 39–100) in the BTT group and 40 min (IQR 24–76) in the ATT group, *p* < 0.001. The admission-surgery time interval was 116 min (IQR 63–214) in the BTT group and 52 min (IQR 24–76) in the ATT group, *p* < 0.001. Mortality in the BTT group was 18.1% and 13.1% in the ATT group. Adjusted OR was 0.406 (0.215–0.789) *p* = 0.006

**Conclusions:**

A trauma team conformation in a war-influenced middle-income country is feasible and reduces mortality as well as admission-surgery and admission-tomography time intervals in trauma patients.

## Introduction

Trauma is a condition with a high burden of morbidity and mortality in the world, causing more than 5 million deaths per year, corresponding to 9% of global mortality [1, 2]. Other authors even consider it as an epidemic, being the main cause of death in adults under 45 years, causing more deaths than tuberculosis, HIV, and malaria together [1, 3]. In Colombia, trauma has reached mortality rates around 6% in the last 10 years. Notably, Cali is considered the third largest city in the country, with a homicide rate of 65 per 100,000 inhabitants by 2015 [3].

The polytrauma patient represents a diagnostic and therapeutic challenge for those involved in their initial care. Given the type of injuries presented by these patients and the high risk of death, a timely diagnosis of life-threatening injuries is of paramount importance. Trauma reference centers must have trained personnel to provide the required care promptly, from the initial approach to the transfer to diagnostic imaging and surgery. In response to this need, in 1922, the American College of Surgeons created the Trauma Committee which provided the basis for the creation of Trauma Teams (TTs) during the first world war. These TTs were multidisciplinary organized responses to provide the necessary care to those wounded in combat fields [4]. Afterward, these TTs transferred to the civil sphere, and in several studies, they have achieved a positive effect in reducing diagnostic delays and the making of therapeutic decisions, which has been shown to reduce the burden of mortality and associated morbidity in trauma patients [5–9].

Regarding the impact of TTs on mortality reduction, authors such as Celso et al. have documented that establishing a trauma care system could reduce 15% of mortality associated with severe trauma [4]. Additionally, Cheng-HsienWang reported a difference in the transfer times to the operating room of 170 min vs. 534 min, which was associated with lower mortality in the multivariate analysis with an odds ratio of 0.34 [10].

Our institution is a level I university hospital for trauma, a tertiary referral center in southwestern Colombia, a middle-income third world country. Since September 2015, a trauma team was established. Its activation, directed by the emergency physician, consists of the assessment of the patient immediately by trauma surgeon and the immediate availability of respiratory therapy, blood bank, portable radiography equipment, operating room, and/or tomography if necessary.

The goal of this study is to analyze the impact of the implementation of a trauma team on the time since the arrival of the patient to the emergency department until the performance of a computed tomography and/or surgery as well as mortality in trauma patients.

## Methods

This is a retrospective analytical study of a historical cohort approved by the Ethics Committee of the Fundacion Valle del Lili. Patients in the ATT (after trauma team) group consisted of those registered in the institutional electronic database from September 1, 2015, to December 2017. Patients in the BTT (before trauma team) group were filtered by trauma diagnosis in the electronic system for recording clinical records of the institution. No exclusion criteria were used. Clinical information was collected from electronic medical records. Demographic, clinical, and outcome variables such as admission-tomography time interval, admission-surgery time interval, and mortality were recorded in the institutional platform of the clinical research center (BDClinic). An exploratory analysis of 10% of the database was performed verifying and correcting the missing data. The normality of the continuous variables was analyzed with the Shapiro-Wilkinson test, and according to the result, they were presented with means and standard deviation or median and interquartile ranges. Categorical variables were presented as absolute numbers and percentages. Subjects of the ATT group were compared with the BTT group. The categorical variables were compared using either a chi-squared or Fisher test, as indicated, and the continuous variables using a Student *t* test or the Wilcoxon-Mann-Whitney test, according to its normality. The mortality of the two groups, stratified by ISS categories, was compared using the Mantes-Haenszel technique.

An explanatory model of the change in mortality with multivariate logistic regression was performed, including the variables that showed a *p* < 0.1 in the univariate comparisons.

Statistical analyses were performed with the Stata 14.1 program (StataCorp. 2015. Stata Statistical Software: Release 14. College Station, TX: StataCorp LP).

## Results

The final sample consisted of 464 patients, 220 for the BTT group and 244 for the ATT group. The median age was 29 and 28 years respectively, 86% males in the BTT group and 84% in the ATT group. Penetrating trauma occurred in 61% of patients in the BTT group and 44% in the ATT group. The median Injury Severity Index (ISS) was> 15 in both groups; however, patients in the BTT group had a lower ISS of 16.5 (RIC 9–34) compared to the ATT group with an ISS of 19 (RIC 11–26). The median heart rate in the BTT group was 90 bpm (RIC 76–110) and 101 bpm (RIC 82–117) in the ATT group (Table 1).

**Table 1:**
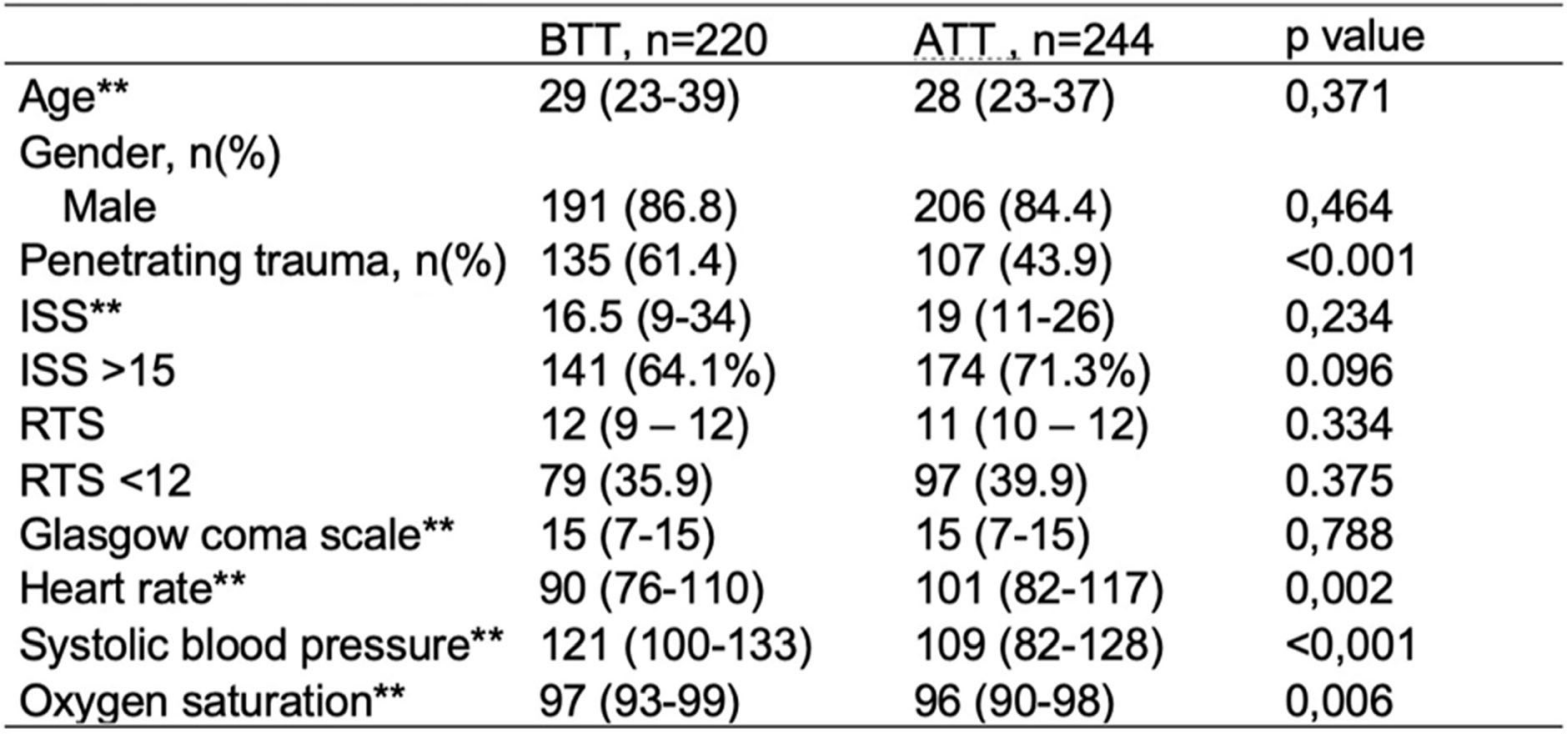
Impact of the implementation of a trauma team. General characteristics

*CT* computed tomography, *ICR* interquartile range, *BTT* before trauma team, *ATT* after trauma team

* Median (ICR)

** Wilcoxon-Mann-Whitney

The admission-tomography time interval in the BTT group was 56 min (RIC 39–100) and 40 min (RIC 24–76) in the ATT group with a *p* < 0.0001 (Tables 2 and 3). The admission-surgery time interval was 116 min (RIC 63–214) in the BTT group and 52 min (RIC 24–76) in the ATT group with a *p* < 0.001. Mortality in the BTT group was 18.1% and 13.1% in the ATT group with a *p* 0.132 (Table 4). However, in the mortality analysis adjusted by the severity of trauma, the reduction in mortality was statistically significant (*p* 0.03) in those patients with an ISS ≥ 16.

**Table 2:**
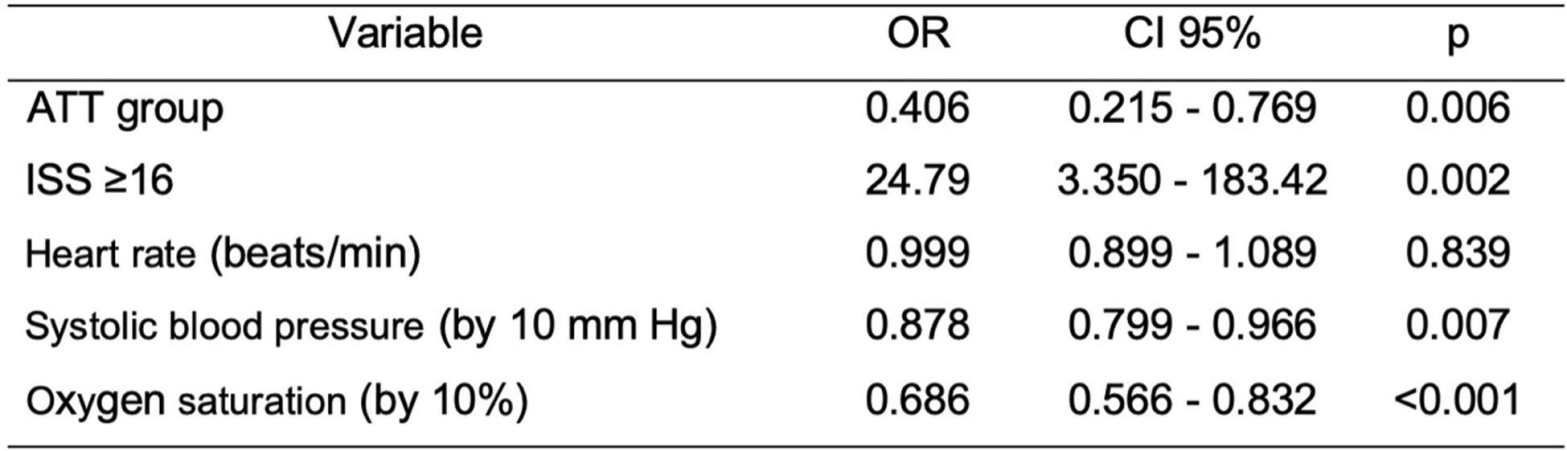
Multiple logistic regression for risk factors of mortality

AUROC 0.860 (0.817–9.04), Hosmer-Lemeshow *p* = 0.684

**Table 3:**
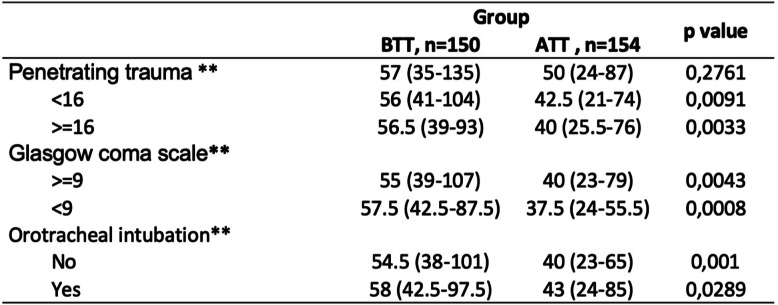
Time to tomography by subgroups

*BTT* before trauma team, *CI* confidence interval

* Median (RIC)

** Wilcoxon-Mann-Whitney

**Table 4:**
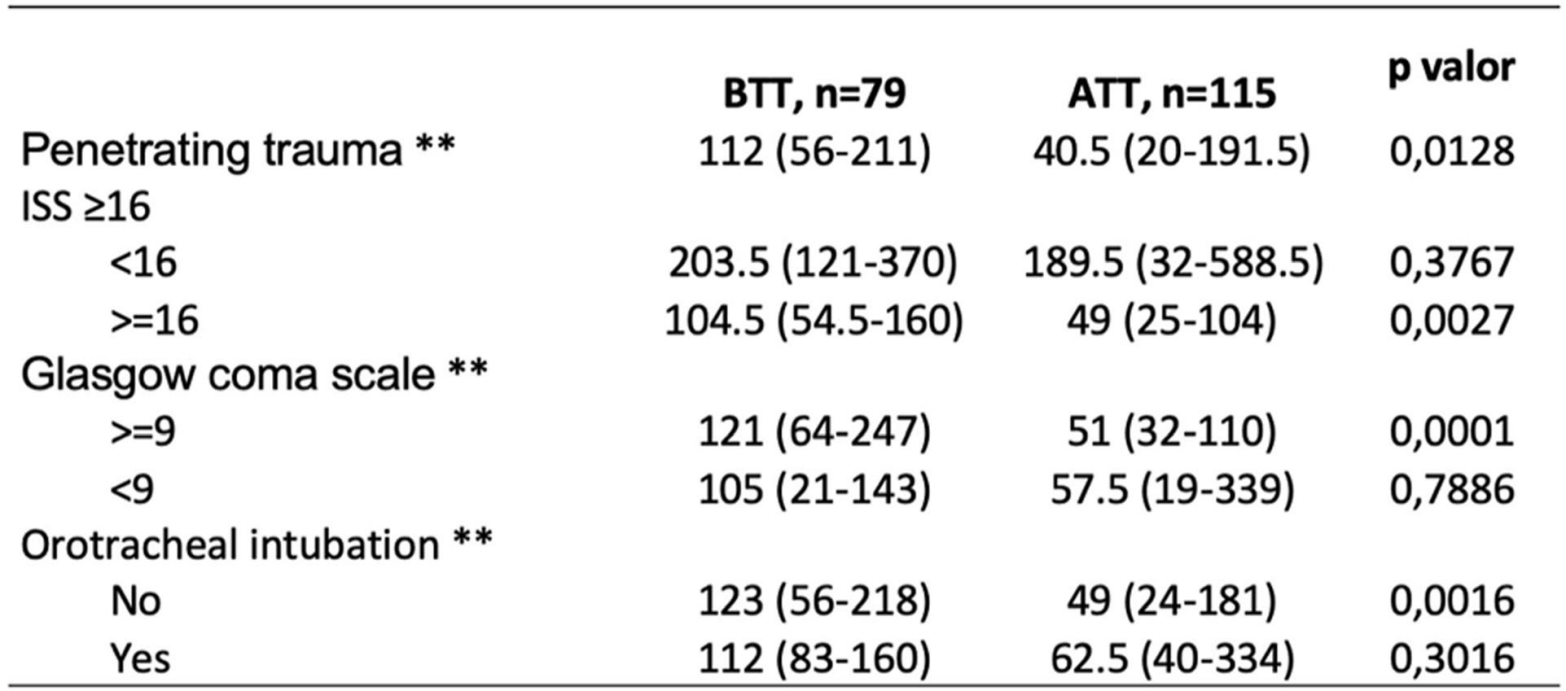
Time to surgery by subgroups

*BTT* before trauma team, *CI* confidence interval

* Median (RIC)

** Wilcoxon-Mann-Whitney

In the subgroup analysis, admission-tomography time interval in patients with an ISS < 16 was reduced from 56 min (IQR 41–104) to 42.5 min (IQR 21–74) with a *p* 0.0091, and in the group with ISS ≥ 16, the admission-tomography time interval was reduced from 56.5 min (RIC 39–93) to 40 min (RIC 25.5–76) with a *p* 0.0033. In those patients with a Glasgow > 9, the admission-tomography time interval was reduced from 55 min (RIC 39–107) to 40 min (RIC 23–79), and in those with Glasgow < 9, the admission-tomography time interval was reduced from 57.5 min (RIC 42.5–87.5) to 37.5 min (RIC 24–55.5).

In the admission-surgery time interval by subgroups patients with an ISS < 16, the time interval was reduced from 203.5 min (RIC 121–370) to 189.5 min (RIC 32–588.5), and in the group ISS > 16, the time substantially reduced from 104.5 min (RIC 54.5–160) to 49 min (RIC 25–104). In those patients with GCS > 9, the time interval was reduced from 121 min (RIC 64–247) to 51 min (RIC 32–110) and patients with GCS < 9 reduced from 105 min (RIC 21–143) to 57.5 min (RIC 19–339).

Patients with an ISS ≥ 16 had an OR 24.79 of death (Table 2). Finally, the results showed that belonging to the ATT group, whose management had been carried out by a trauma team, had an OR 0.4 of death when compared to those managed without a trauma team.

## Discussion

Developing a trauma team is challenging, especially in a middle-income country going through an internal war against guerillas. Time to tomography and time to surgery were chosen as indirect variables to measure optimal patient care. In the trauma scenario, fastest is better. This is due to the potentially deadly injuries patients suffer and taking into account the premise that the quicker you act, the higher the chances of survival are going to be. Plus, several studies mentioned above have taken these two variables (time to surgery and time to tomography) as a measure to indicate improvement in patient care. The admission-tomography and admission-surgery time interval in polytrauma patients decreased with the implementation of a TT, being statistically significant and clinically relevant. The time to tomography interval decreased by 26%, reducing the median by 16 min (*p* 0.0001). The time to surgery interval was reduced to a greater extent, decreasing by 56%, or 64 min (*p* 0.0009). Similarly, mortality was significantly reduced by 9.4% (*p* 0.03) in patients with ISS > 15.

Both groups (BTT and ATT) are demographically similar, with a greater proportion of male patients in their early thirties. There was a 15% reduction in penetrating trauma, possibly due to a relative decrease in rural violence resulting from the demobilization of a guerrilla group in the country.

Establishing specific criteria to activate a TT reduces the number of unnecessary TT activations that do not need a team response. This is one of the reasons why our results evidenced a difference in ISS and vital signs before a TT was created, where no criteria was used to respond upon trauma patients, and after a TT was implemented. The median ISS of the ATT group was higher. This effect is expected given that, in this group, previously established criteria was used to activate the trauma team based on clinical and injury variables (Table 5), meaning that those patients were more severely injured. ATT patients were more hypotensive and had more tachycardia. This is important in the implementation of trauma teams since one of the main objectives is to identify those severely injured patients who will benefit from a trauma team.

**Table 5:** Trauma Team Activation Criteria

Intubated patient upon arrival to the emergency department	
Dyspnea or respiratory failure	
Hypotension SBP < 90 mmHg	
Glasgow Coma Scale <9	
Penetrating trauma in the trunk	
Traumatic limb amputation	
Unstable pelvic fracture	
Previously operated trauma patient	
2 or more trauma patients at the same time	
Emergency physician criteria	

Trauma team criteria activation at Fundación Valle del Lili, Cali, Colombia

In patients with ISS ≥ 16, mortality was significantly reduced. This is possibly because minor trauma (ISS < 16) does not require a multidisciplinary emergent intervention, and it could cause extra cost.

In the subgroup analysis, according to clinical characteristics, a significant decrease in the time to tomography and surgery interval was evident in all groups, which implies that both in severely ill patients and those less compromised, a TT reduces attention times. However, there was a greater decrease in the admission-tomography time interval in those patients with GCS < 9, while in the admission-surgery interval, the group with the shortest time was those with severe trauma (ISS > 16) from 104 min to 49 min possibly related for both groups with the severity of the injury. Although a TT managed to reduce the time intervals to both surgery and tomography in all patients, it is in the severely ill (ISS > 16) patients in which reducing the time of attention impacts mortality.

Development of a TT is challenging, and human and physical resources are indispensable in achieving favorable results. The path to the implementation of a TT in our institution has been tough due to several social, economic, and infrastructure barriers. In Colombia, TTs are scarce; few medical care facilities have established a TT. In our region (southwest Colombia), we are the first, and only, established TT. One of the biggest barriers to the establishment of this group includes the internal war in our country which is generated by two main guerrillas and more than a dozen criminal city groups. Due to the abundance of armed groups, trauma is very common in our institution, with an average of two severe (ISS > 16) cases of trauma per day, usually penetrating. This number of patients requires a large amount of economic and physical resources, leading to the second barrier due to the fact that we are a middle-low-income country. Finally, a third barrier is the dispersion of our rural community as patients usually live at least an hour away from our institution leading to a delay in the transportation and timely management of patients.

We have accomplished important goals while developing this TT in our population. First of all, we replicated our model of TT response in the pediatric population who are also victims of the war civil and military war in our country. Studies are running to evaluate its effect on this population. Nevertheless, we are also traveling to rural hospitals to talk about our experience managing trauma and helping small institutions to develop trauma teams. Last but not the least, one of the discussions right now in our trauma team is getting to determine which variables are the best variables to predict an adequate TT activation. We have a study running right now evaluating the predictable value of each one of our TT activation criteria, which indeed is a globally debated subject.

## Conclusion

Trauma teams have been shown to be of great benefit for the multidisciplinary attention for trauma teams in terms of attention times and mortality. A trauma team conformation in a war-influenced middle-income country is feasible and reduces mortality as well as admission-surgery and admission-tomography time intervals in polytrauma patients.

## Data Availability

Please contact author for data requests.
